# Association Between Self-Reported Climacteric Symptoms and Pain Intensity: A Cross-Sectional Study of 6000 Japanese Men and Women

**DOI:** 10.3390/healthcare14213595

**Published:** 2026-10-23

**Authors:** Yukiko Shiro, Shinsuke Inoue, Young-Chang Arai, Koichi Shinohara, Tatsunori Ikemoto

**Affiliations:** 1 Department of Rehabilitation Sciences, Nagoya Gakuin University, Nagoya 456-0062, Japan; 2 Department of Pain Medicine, School of Medicine, Aichi Medical University, Nagakute 480-1195, Japan; arainon@aichi-med-u.ac.jp; 3 Kaguyama Orthopedic Clinic, Nisshin 470-0134, Japan; sinoue0626@gmail.com; 4 Department of Obstetrics and Gynecology, School of Medicine, Aichi Medical University, Nagakute 480-1195, Japan; sino0114@aichi-med-u.ac.jp; 5 Department of Orthopedics, School of Medicine, Aichi Medical University, Nagakute 480-1195, Japan; tatsunon31@gmail.com

**Keywords:** climacteric symptoms, pain, sex difference

## Abstract

**Highlights:**

**What are the main findings?**

• Climacteric symptoms were positively associated with pain intensity in both men and women, and there was no clear evidence of a sex difference in this association.
• Vasomotor symptoms in women and psychological symptoms in men remained associated with pain after excluding pain-related items; however, formal tests did not show that the psychological association differed by sex.

**What are the implications of the main findings?**

• Climacteric symptoms were associated with pain in middle-aged men as well as women, suggesting that this is not a women-specific issue.
• Prospective studies that measure hormone levels, menopausal status, and pain duration are needed to clarify these associations.

**Abstract:**

**Background/Objectives**: The prevalence of pain increases with age, particularly in middle-aged women, when sex hormone levels fluctuate drastically. While climacteric symptoms and their relationship with pain have been extensively studied in women, it remains unclear whether these symptoms and their association with pain are limited to women. This study aimed to investigate the association between climacteric symptoms and pain in both women and men. **Methods**: A cross-sectional online survey using a non-probability quota panel conducted in March 2024 assessed the presence, location, and intensity of pain during the past 28 days (numerical rating scale ≥ 1, irrespective of pain duration) among 6000 Japanese adults aged 30–59 years (3000 women, 3000 men). Climacteric symptoms were assessed using the Simplified Menopausal Index for women and the Aging Males’ Symptoms scale for men. Quality of life was assessed using the EQ-5D-5L. Pain intensity was analyzed only in participants with pain (433 women, 488 men). **Results**: As symptom scores increased, both men and women showed higher pain prevalence and intensity and lower quality of life. Symptom scores were positively correlated with pain intensity in both sexes (Spearman’s ρ = 0.35 in women, 0.39 in men), with no evidence of a sex difference (interaction *p* = 0.991). Somatic scores showed the strongest associations (standardized β = 0.21 in women, 0.22 in men), followed by vasomotor symptoms in women (β = 0.16) and psychological symptoms in men (β = 0.20). The associations remained after excluding pain-related items. **Conclusions**: Climacteric symptoms were associated with pain intensity in both men and women, without clear evidence of a sex difference. Causality cannot be inferred from this cross-sectional design; prospective studies are needed.

## 1. Introduction

The prevalence of chronic pain increases with age, and chronic musculoskeletal pain is common in people in their 30s to 50s [1,2]. Significant changes in sex hormone levels during midlife (the climacteric transition) are associated with various physical and psychological symptoms, including pain. Specifically, musculoskeletal pain, joint pain, headache, and lower back pain are the most frequently reported pain-related climacteric symptoms [3]. In women, a rapid decline in estrogen levels during menopause may affect pain sensitivity. Estradiol—the most potent form of estrogen—has also been linked to musculoskeletal pain, stiffness, and mood disturbances during this period [4]. Estrogen exerts both pain-increasing and pain-relieving effects, and its role in pain modulation is complex. Thus, no definitive causal relationship between estrogen deficiency and pain has been established [5,6,7].

Hormonal changes are thought to contribute to menopause-related discomfort, whereas aging-associated physiological changes may contribute to increased pain. For example, many factors, including senescence-related secretory phenotypes, low reactivity of chondrocytes to growth factors, mitochondrial dysfunction, oxidative stress, and abnormal accumulation of advanced glycation end products, may play key roles in the pathogenesis of age-related osteoarthritis [8]. Hence, pain experienced during this period may not be exclusively driven by hormonal fluctuations.

Notably, men also undergo sex hormone changes with age, although these changes are less commonly recognized. In contrast to the relatively rapid decline in estrogen levels in women, men experience a gradual decrease in testosterone levels with age [9,10]. Testosterone deficiency is associated with symptoms, including sexual dysfunction, mood disturbances, and physical changes [11]. However, its diagnosis requires both consistent symptoms and unequivocally low serum testosterone concentrations, and in a European population-based study, only sexual symptoms showed a syndromic association with low testosterone levels [12,13]. Terms such as andropause, male menopause, partial androgen deficiency of the aging male, and late-onset hypogonadism are used to describe this condition, but the relationship between age-related testosterone decline and these symptoms remains controversial, and only a small fraction of older men are reported to have this syndrome [14]. Nevertheless, the relationship between climacteric symptoms and pain in men is poorly understood, with fewer studies available compared with those on menopause in women.

Some studies suggest that low testosterone levels may be associated with reduced activation of brain regions involved in descending pain modulation, which could contribute to chronic pain states [15,16]. Testosterone has also been reported to have antinociceptive effects, reducing hyperalgesia in both men and women [17], and higher testosterone levels have been suggested to be associated with a lower risk of widespread and chronic musculoskeletal pain [16]. However, these findings come mainly from experimental and observational studies, and whether age-related changes in testosterone contribute to pain in men remains unclear.

Consequently, we hypothesized that the relationship between climacteric symptoms and pain is not exclusive to women. Accordingly, this study aimed to investigate the association between self-reported climacteric symptoms and pain of any duration during the past 28 days (numerical rating scale ≥ 1) in both women and men, aged 30–59 years.

## 2. Materials and Methods

A cross-sectional online survey on the relationship between pain, climacteric symptoms, and sex-related differences involving 6000 Japanese adults aged 30–59 years (3000 women and 3000 men) was conducted from 5 to 25 March 2024.

The age range of 30–59 years was chosen because chronic musculoskeletal pain is most prevalent among Japanese adults in their 30s to 50s [1,2] and to include the period before and after the typical age at menopause in women. The same range was applied to men for comparability. Menopausal status was not assessed; many participants in their 30s are therefore not expected to be perimenopausal, and participants in their 30s were included as a comparison group with, on average, fewer climacteric symptoms.

Participants were recruited through the website of an Internet research firm, Cross Marketing Inc. (Tokyo, Japan). The survey panel was a non-probability (opt-in) online panel, and quota sampling was used to obtain 500 individuals per 5-year age group for both men and women. The research firm did not disclose the number of invitations and incomplete responses, or the response rate, because these figures are confidential customer information. Responses that did not pass the research firm’s data quality checks (*N* = 753) were excluded, and data collection continued until the quota of 6000 valid responses (3000 women and 3000 men) was reached. The survey was closed: only registered panel members could respond, and the research firm verified panel IDs to prevent multiple responses.

The standard error of the mean decreases with an increase in the sample size, and a sample size of approximately 400–500 yields a standard error small enough to provide an approximately 5% margin of error for estimating proportions within each sex and age group [18]. No formal power calculation was performed for the correlation and regression analyses. The initial screen presented the study’s purpose, its scope, and details regarding the publication of the results. Participants were also informed about their right to withdraw at any time without any consequences. To proceed, they were required to carefully review the information and indicate their informed consent by selecting a confirmation button. Only those who provided consent were granted access to the subsequent survey screen. The survey form was pretested, and its technical functionality was checked before data collection. Items were presented in a fixed order, and respondents could not proceed to the next item without answering. The authors received fully anonymized data. The participants were given online shopping points as a reward equivalent to $5. The survey protocol was approved by the Nagoya Gakuin University Research Ethics Committee (No. 2023-24; approved on 4 March 2024).

### 2.1. Assessment

A self-administered online survey collected the following information: (1) participant characteristics (sex, age, weight, and height), (2) pain and its location, (3) climacteric symptoms, and (4) health-related quality of life (HRQOL).

Participants were asked if they had experienced pain during the past 28 days, and mean pain intensity and location of the pain were recorded for those who reported experiencing pain. Pain locations were recorded using a checklist. Subjective pain severity was rated on an 11-point Numerical Rating Scale (NRS), where 0 = no pain and 10 = worst imaginable pain. Individuals who reported a pain rating of 1 or greater on the NRS were assigned to the pain group to effectively understand the impact of pain as a possible contributing factor to physical discomfort. The duration, cause, and type of pain were not assessed; thus, pain in this study includes acute, recurrent, and chronic pain.

The climacteric symptoms were assessed using the Simplified Menopausal Index (SMI) for women and the Aging Males’ Symptoms (AMS) scale for men. The SMI consists of 10 questions evaluating vasomotor symptoms (four items: hot flashes, sweats, chills, shortness of breath, or palpitations), psychological symptoms (four items: sleep disturbances, easy excitability or irritability, worry about self-depression, and headache, vertigo, or nausea), and somatic symptoms (two items: easy fatigability and shoulder stiffness, lumbago, or joint pain). Total scores of 0–25, 26–50, and 51–100 represent “no,” “mild,” and “severe” problems, respectively [19,20]. The SMI is a simple 10-item index that has been used in Japan to assess climacteric symptoms in women [19,20]; the three symptom groups used in this study correspond to the physical (items 1–4), mental (items 5–8), and general (items 9–10) symptoms described previously [20]. The AMS scale is used to measure physical and psychological symptoms associated with aging in men; it does not establish biochemical hypogonadism or testosterone deficiency. Symptoms are generally evaluated by summing the total score and severity points for each subscore (psychological, somatic, and sexual). The severity of symptoms according to the total scores of 17–26, 27–36, 37–49, and ≥50 indicates “none/little,” “mild,” “moderate,” and “severe” symptoms, respectively [21]. In this study, to compare groups of similar size, participants were classified within each sex into quartile groups (Q1–Q4) of the total score. These groups are relative to each sex and do not represent clinical severity.

HRQOL was assessed using the EuroQol-5 Dimensions-5 Levels (EQ-5D-5L) scale, which calculates utility values, quantifying 1 as perfect health and 0 as mortality [22].

### 2.2. Statistical Analysis

Data on participant characteristics are presented as median (first and third quartiles). Comparisons of pain prevalence and sex differences were performed using the χ^2^ test or Mann–Whitney U test. Prevalence is presented with 95% CIs calculated using the Wilson score method. As climacteric symptom measures differ between sexes and groups according to symptom score quartiles, the Kruskal–Wallis test was used to compare the quartile groups, and the Dunn–Bonferroni post hoc test was used for pairwise comparisons. Because pain intensity and symptom scores are ordinal and skewed, as indicated by histograms and the Shapiro–Wilk test, non-parametric tests were used.

Spearman’s rank correlation coefficient was used to assess the correlation between pain intensity and climacteric symptoms. Analyses of pain intensity were restricted to participants with pain, because intensity was recorded only for them. Multiple regression analysis was performed separately for each sex with pain intensity as the dependent variable and SMI or AMS subscores as independent variables, with age and body mass index as covariates. Unstandardized coefficients (B) with 95% confidence intervals (CIs), standardized coefficients (β), variance inflation factors (VIFs), and R^2^ were reported. Because of the cross-sectional design, the results were interpreted as associations. To check the regression assumptions, we inspected the histogram and normal P–P plot of standardized residuals and the scatterplot of standardized residuals against standardized predicted values, separately for women and men. Multicollinearity was assessed using VIFs.

To compare the associations between sexes, SMI and AMS scores were standardized (z-scores) within each sex using all participants of that sex, because the two scales differ in items, score range, and subscale structure. Sex differences were tested in a pooled model of participants with pain that included sex, the z-score, a sex × z-score interaction term, age, and BMI. The model was fitted for the total score and for the psychological and somatic subscores entered simultaneously, together with the sex-specific vasomotor (women) and sexual (men) scores. Sex-specific slopes were obtained by changing the reference category of sex. Vasomotor and sexual scores were not compared between sexes.

Because the SMI and AMS include items that overlap with pain, we repeated the correlation, regression, and interaction analyses after excluding the items that include pain complaints (SMI: “shoulder stiffness, lumbago, or joint pain” and “headache, vertigo, or nausea”; AMS: “joint pain and muscular ache”). After the exclusion, the SMI psychological subscale consisted of three items and the somatic subscale of a single item (easy fatigability).

All survey items were mandatory, and a response could not be submitted with unanswered items; therefore, the final dataset had no missing data and no imputation was required. The research firm applied data quality checks and excluded responses with identical answers across all items, extremely short response times, and implausible entries (e.g., a height of 300 cm).

Statistical significance was set at *p* < 0.01 for multiple comparisons in all analyses. The analyses were considered exploratory, and effect sizes and 95% CIs were emphasized. This study was reported according to the STROBE and CHERRIES checklists. Statistical analyses were conducted using SPSS version 26 (IBM, Armonk, NY, USA).

No sampling weights were applied; the results describe the study sample and not the Japanese population.

## 3. Results

### 3.1. Participant Characteristics and Pain Prevalence

Table 1 presents the participant characteristics according to sex in the groups with and without pain. The prevalence of pain was 14.4% (95% CI 13.2–15.7%; n = 433) and 16.3% (95% CI 15.0–17.6%; n = 488) in women and men, respectively. The difference in prevalence between sexes was not statistically significant at the pre-specified level (*p* = 0.049). Pain intensity was significantly higher in women than in men (median 6 vs. 5), though the difference was small (*p* = 0.002). The EQ-5D-5L utility value did not differ between sexes (*p* = 0.057). Climacteric symptoms based on SMI and AMS scores were significantly higher in both men and women in the pain group. Because of the quota sampling from a non-probability panel, these prevalences are not estimates for the Japanese population. See Supplementary Table S1 for data from the SMI and AMS with pain-related items excluded.

### 3.2. Climacteric Symptom Score Quartiles and Pain Prevalence

**Figure 1:**
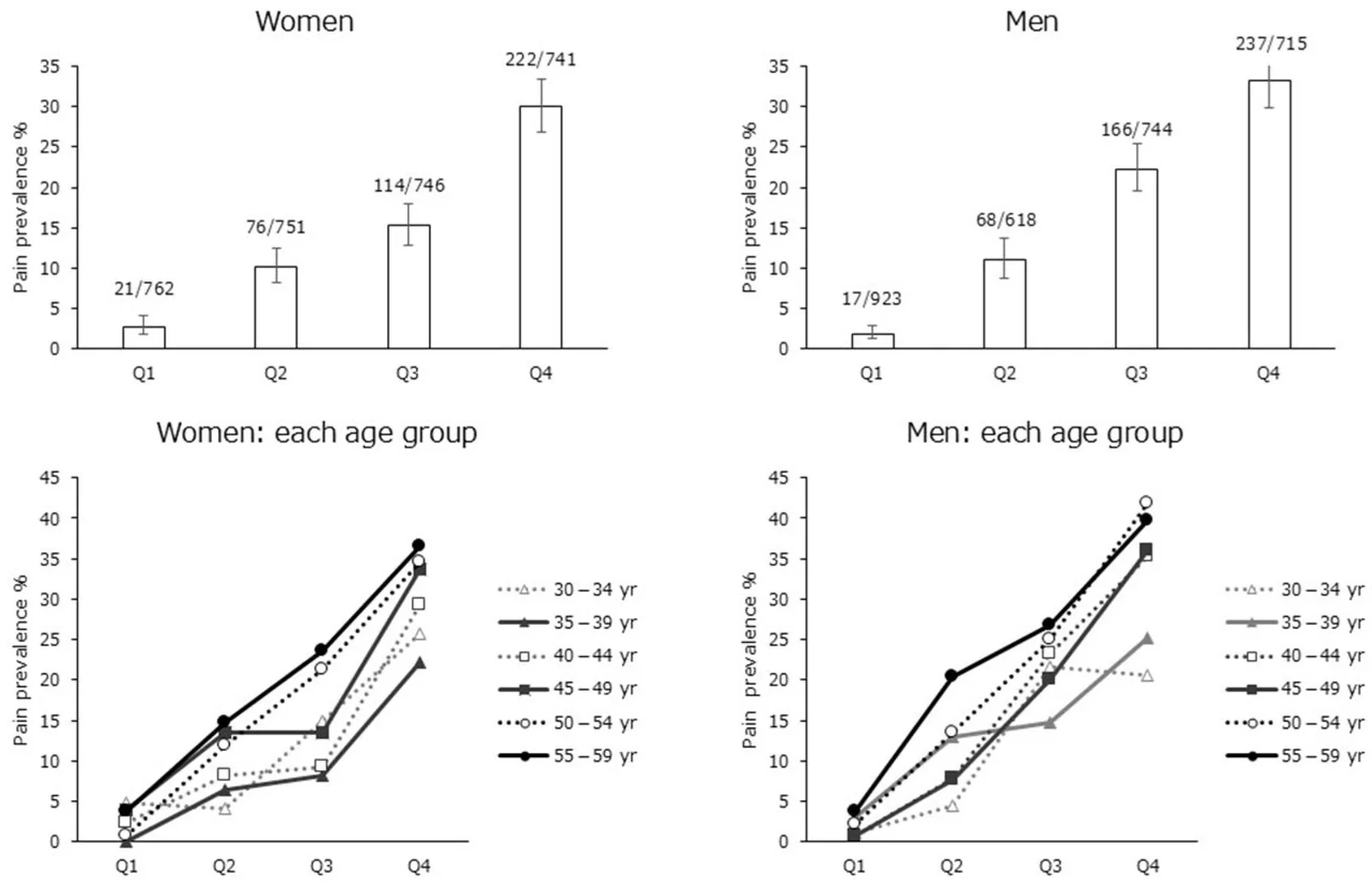
Climacteric symptoms and pain prevalence. Bars show the percentage of participants with pain in each quartile group (Q1–Q4) of the Simplified Menopausal Index (women) and the Aging Males’ Symptoms scale (men). Error bars indicate 95% confidence intervals (Wilson score method), and the number of participants with pain/total number (n/N) is shown above each bar. Quartile groups are defined within each sex and do not indicate clinical severity. The lower panels show the prevalence in each 5-year age group (without confidence intervals; see Supplementary Table S2).

**Figure 2:**
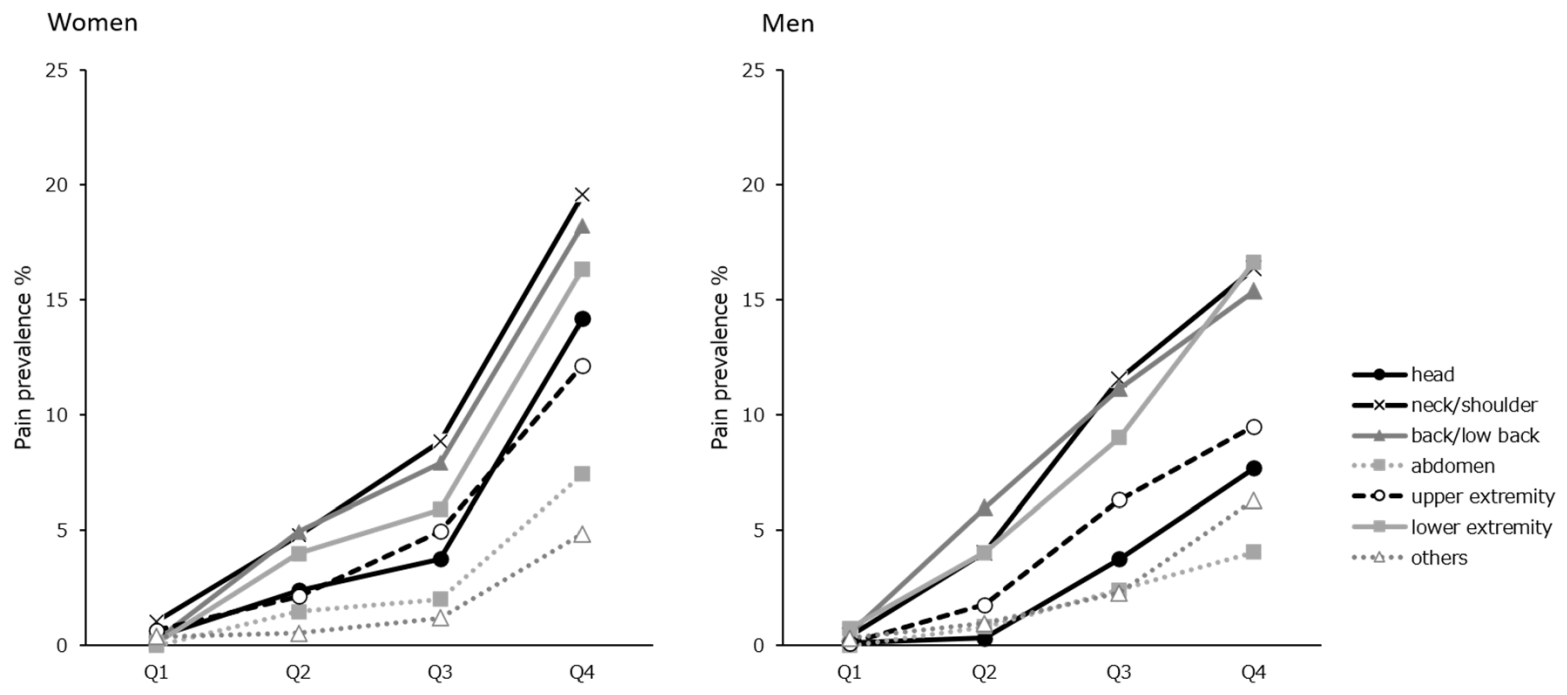
Change in pain location associated with climacteric symptoms. Lines show the percentage of all participants in each quartile group who reported pain at each site; participants could report more than one site. Numbers and 95% confidence intervals are given in Supplementary Table S2 (Panel B).

The SMI and AMS total scores were divided into quartile groups within each sex as follows: 0–12 and 17–18, Q1 (lowest); 13–30 and 19–24, Q2; 31–49 and 25–36, Q3; and 50–100 and 37–85, Q4 (highest), respectively. As climacteric symptoms increased from Q1 to Q4, the prevalence of pain increased in both sexes (Figure 1): from 2.8% (95% CI 1.8–4.2%) to 30.0% (26.8–33.4%) in women and from 1.8% (1.2–2.9%) to 33.1% (29.8–36.7%) in men. A similar pattern was observed in all age groups for both sexes when investigated separately for each age group at 5-year intervals, with the lowest prevalence in Q1 in every age group (Figure 1, Supplementary Table S2); the pattern was less clear in men aged 30–34 years (Q3 vs. Q4). However, the number of participants per cell was small, and we did not formally test whether the association differed by age. Furthermore, pain prevalence at each site increased with the quartile group in both men and women, with no particular increase at a specific pain site (Figure 2, Supplementary Table S2).

### 3.3. Symptom Score Quartiles and Pain Intensity, Location, and HRQOL

**Figure 3:**
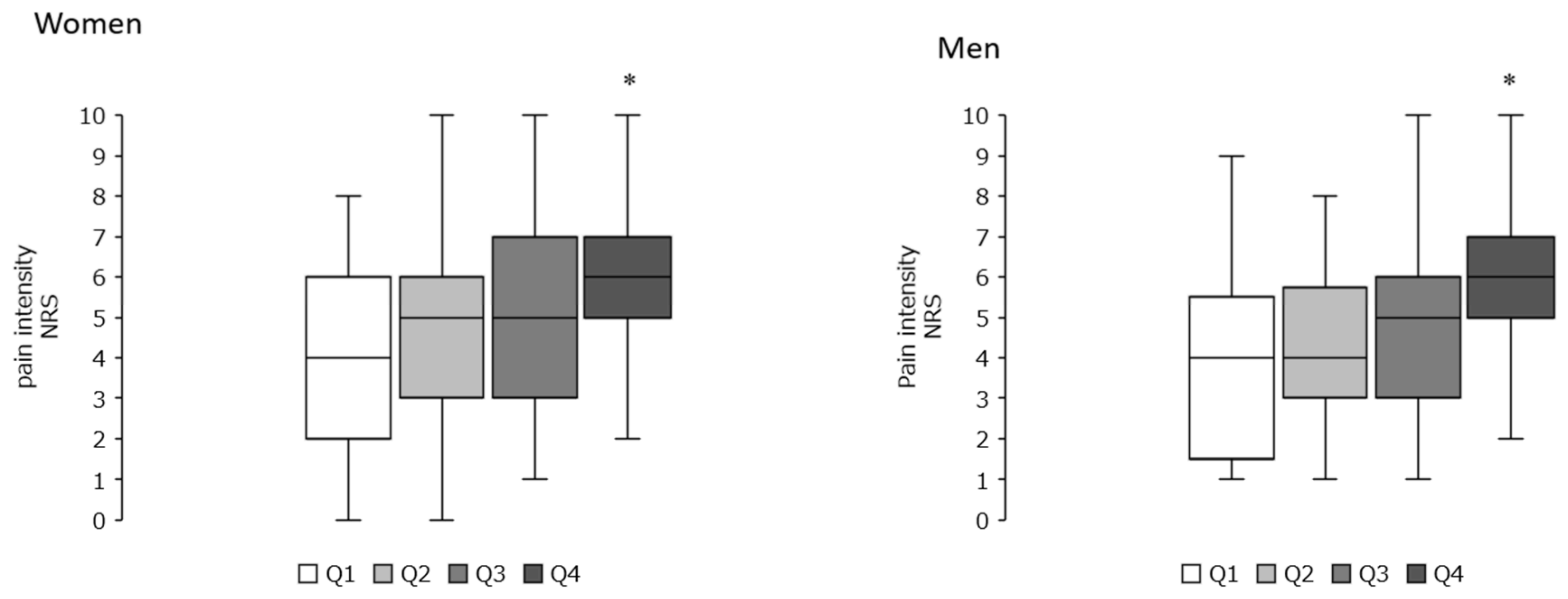
Climacteric symptoms and pain intensity. *, vs. Q1–Q3: adj. *p* < 0.01.

**Figure 4:**
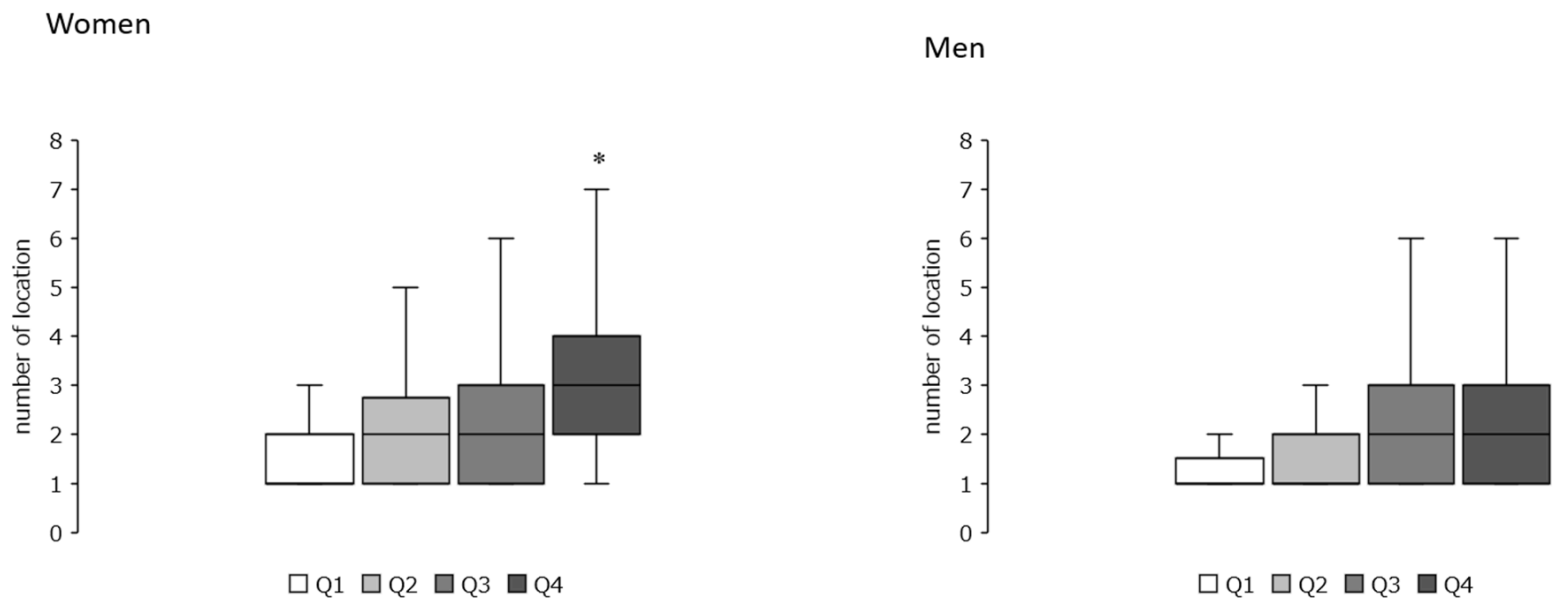
Climacteric symptoms and number of pain locations. *, vs. Q1–Q3: adj. *p* < 0.01.

**Figure 5:**
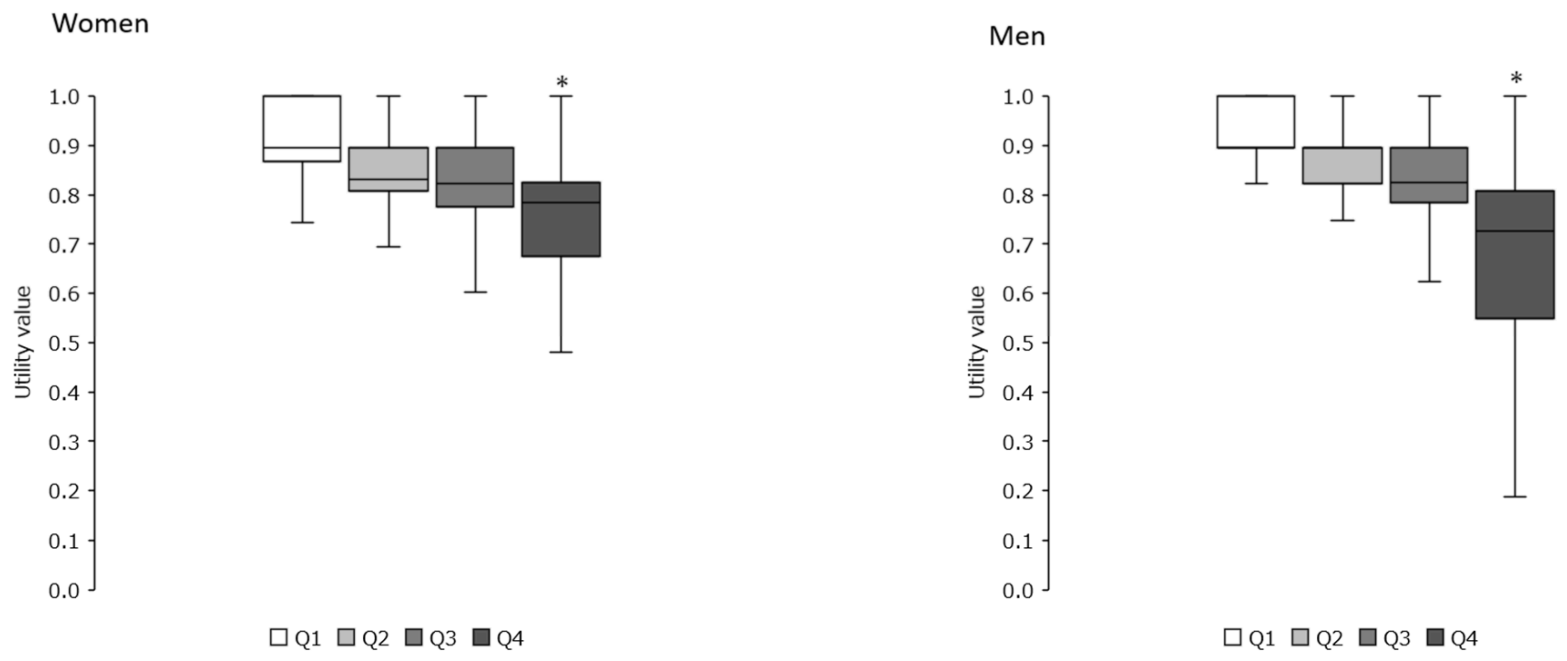
Climacteric symptoms and EQ-5D-5L. *, vs. Q1–Q3: adj. *p* < 0.01.

The Q4 group reported the highest pain levels for both men and women when pain intensity was compared by quartile group (Figure 3). Similarly, women in the Q4 group reported significantly more pain locations than those in the Q2 or Q3 groups, whereas this difference was not significant in men (Figure 4); whether this pattern differed by sex was not formally tested. Furthermore, we compared pain symptoms between men and women according to the quartile group (Table 2). Pain prevalence was higher in men than in women in the Q3 group (*p* = 0.001). Pain intensity did not differ significantly between women and men in any quartile group (all *p* ≥ 0.020). The number of pain locations affected was greater in women than in men in the Q4 group (*p* < 0.001). The utility values of EQ-5D-5L (which include a pain/discomfort dimension) were significantly lower in the Q4 group than in the Q1, Q2, or Q3 groups, in both men and women (Figure 5).

### 3.4. Association Between Climacteric Symptoms and Pain Intensity

Among participants with pain (433 women and 488 men), all SMI and AMS total scores and subscores demonstrated significantly positive correlations with pain intensity (Table 3). SMI and AMS both showed a correlation with pain intensity even when pain-related items were excluded (Supplementary Table S3). Multiple regression analysis was performed separately for each sex to examine the associations between climacteric symptoms and pain intensity, with pain intensity as the dependent variable and SMI or AMS sub-scores as the explanatory variables. Age and body mass index were entered as covariates to adjust for age and physical characteristics. Somatic and vasomotor scores were associated with pain intensity in women (standardized β = 0.208, *p* < 0.001; β = 0.160, *p* = 0.004, respectively), whereas somatic and psychological scores were associated with pain intensity in men (β = 0.224, *p* = 0.002; β = 0.203, *p* = 0.005, respectively) (Table 4). In both sexes, the somatic subscore showed the largest standardized coefficient. The models explained 13.5% (women) and 16.2% (men) of the variance in pain intensity (adjusted R^2^), and all variance inflation factors were below 3.2 (Table 4). In both sexes, residuals were approximately normally distributed, with no clear departure from homoscedasticity or linearity.

### 3.5. Sex Differences in the Associations Between Climacteric Symptoms and Pain Intensity

In a pooled model of participants with pain (n = 921) that included a sex × symptom interaction term and was adjusted for age and BMI, a one-SD higher total symptom score was associated with higher pain intensity in women (B = 0.73, 95% CI 0.54 to 0.91) and in men (B = 0.73, 95% CI 0.58 to 0.88). The sex × symptom interaction was close to zero (B = 0.00, 95% CI −0.23 to 0.24, *p* = 0.991), providing no evidence that the association differed by sex.

When the psychological and somatic subscores were entered simultaneously (together with the sex-specific vasomotor or sexual score, age, and BMI), both subscores were associated with pain intensity in men (psychological: B = 0.35; somatic: B = 0.40), whereas in women the somatic subscore was associated with pain intensity (B = 0.52) and the psychological subscore was not (B = 0.15; Table 5). However, neither the sex × psychological interaction (B = 0.20, 95% CI −0.14 to 0.54, *p* = 0.246) nor the sex × somatic interaction (B = −0.12, 95% CI −0.50 to 0.27, *p* = 0.546) was statistically significant. Vasomotor symptoms (women only) and sexual symptoms (men only) have no counterpart in the other sex and were not compared between sexes. Thus, the data do not provide clear evidence that these associations differ by sex, and differences in statistical significance between the sex-stratified models (Table 4) should not be interpreted as sex differences.

### 3.6. Sensitivity Analyses Excluding Pain-Related Items

After excluding the items that include pain complaints (SMI: “shoulder stiffness, lumbago, or joint pain” and “headache, vertigo, or nausea”; AMS: “joint pain and muscular ache”), the association between the total score and pain intensity remained (women: B = 0.66, 95% CI 0.48 to 0.84; men: B = 0.71, 95% CI 0.56 to 0.85; per 1-SD increase). The sex × symptom interaction was again close to zero (B = 0.04, 95% CI −0.19 to 0.27, *p* = 0.718) and similar to that for the original total scores, providing no evidence that the association differed by sex (Supplementary Table S4).

In the subscore regression models, vasomotor symptoms in women (β = 0.193, *p* < 0.001) and psychological symptoms in men (β = 0.238, *p* = 0.002) remained associated with pain intensity, whereas the associations for the somatic subscores were attenuated (women, fatigue item only: β = 0.121, *p* = 0.035; men: β = 0.166, *p* = 0.031) (Supplementary Table S5).

## 4. Discussion

To the best of our knowledge, this study is among the first to investigate the association between the characteristics of climacteric symptoms and pain and potential sex-related differences in both women and men. Previous studies have compared pain between women and men according to menopausal status or age, including the prevalence and severity of chronic pain before and after menopause [23], the prevalence of low back pain [24], and lumbar disc degeneration [25], but, to our knowledge, none examined the severity of climacteric symptoms in both sexes.

The results of this survey involving participants in their 30s to 50s did not show a clear difference in pain prevalence between men and women (*p* = 0.049, not significant at the pre-specified level). In addition, higher symptom score quartiles were associated with higher pain prevalence in all age groups examined, and lower HRQOL in both men and women. Pain intensity was also higher in the Q4 groups of both sexes. These findings suggest that climacteric symptoms and pain co-occur in both women and men.

The average age of menopause in Japanese women is 48.3 ± 4.8 years, and 80% of women had their menopause between 45 and 54 years of age [26]. Hormonal changes around this age may therefore be one of several possible factors, as may be the case for age-related changes in men: climacteric symptoms such as decreased muscle strength and libido have been reported more frequently in men aged 50 years and older than in those in their 40s [27], and serum testosterone has been reported to decline gradually with age (approximately 1.6% per year for total testosterone in men aged 40–70 years) [9,10]. However, hormone levels and menopausal status were not measured, and we did not test whether the association differed by age; these explanations remain hypothetical.

Pain intensity was higher in women than in men overall (median 6 vs. 5), and women in the Q4 group reported more pain locations than men; whether the association between symptoms and the number of pain locations differed by sex was not formally tested. Generally, women are more sensitive to pain and at a higher risk of clinical pain, particularly chronic pain, than men [28]. Unlike previous studies, this study did not specify the duration, cause, or type of pain, and thus included acute, recurrent, and chronic pain; whether this affected the sex difference in prevalence cannot be tested because pain duration was not recorded. Pain prevalence (14.4% and 16.3%) was lower than the reported prevalence of chronic pain in Japan [1,2], although pain of any duration was included; this may reflect the selection of online panel participants and the definition and wording of the pain question.

Among the subscores, the somatic subscore showed the strongest association with pain intensity in both sexes, followed by vasomotor symptoms in women and psychological symptoms in men (Table 4). However, both the SMI and AMS include questions on pain in the somatic subscale, so the association may be partly circular; indeed, the associations for the somatic subscores were attenuated after excluding pain-related items, whereas those for vasomotor symptoms in women and psychological symptoms in men remained (Supplementary Table S5). Formal interaction tests did not show that the associations of the psychological or somatic subscores differed by sex (Table 5). Age was not associated with pain intensity after adjustment among participants with pain (Table 4).

At some time point during the climacteric transition, 80% of women experience classic climacteric vasomotor symptoms, such as hot flashes or flushes and night sweats. Subsequently, vasomotor symptoms can worsen psychological symptoms, ultimately decreasing the quality of life [29]. Changes in estrogen levels may lead to the development of vasomotor symptoms.

Estrogen also affects sensory and pain systems. Notably, estrogen replacement has been reported to exert an early antinociceptive effect on mechanical hyperalgesia in ovariectomized rats, although the effect depended on the time course of estrogen change [30]. Additionally, 17-beta estradiol can reduce neuropathic pain in rats [31]. Meanwhile, estrogen has also been reported to increase hyperalgesia [32], indicating its complex pain-modulating role. In the present study, the severity of the vasomotor symptoms due to decreased estrogen levels was associated with pain intensity, suggesting that changes in estrogen levels may be related to pain in women. However, hormone levels were not measured, and the design was cross-sectional; therefore, this interpretation remains hypothetical, and findings from animal models cannot be directly extrapolated to women.

Testosterone plays a pivotal role in maintaining balance within the multidimensional psychological network of mood, behavior, self-perception, and perceived quality of life in men at any age [33]. Furthermore, testosterone exerts antinociceptive effects by limiting the production of inflammatory cytokines such as tumor necrosis factor-α and by reducing signaling through capsaicin receptors in dorsal root ganglion neurons [34]. Based on these findings, it is possible that psychological symptoms such as low mood, anxiety, and pain may be linked to decreased testosterone levels; however, testosterone was not measured in this study, and this interpretation remains speculative. The AMS is a symptom scale and cannot diagnose testosterone deficiency, which requires consistently low serum testosterone concentrations in addition to symptoms [13]; moreover, in a large European population study, only sexual symptoms showed a syndromic association with low testosterone, whereas psychological and physical symptoms were less specific [12]. Pain may also be influenced by psychosocial factors. Whether psychological changes due to changes in sex hormone levels affect pain in middle-aged men, or whether psychosocial factors unrelated to sex hormones do so, remains unclear. The association between psychological symptoms and pain intensity in men persisted after excluding pain-related items (Supplementary Table S5).

Reverse causation and bidirectionality should also be considered. Pain may worsen fatigue, sleep disturbance, and low mood, which can in turn raise SMI and AMS scores, and these factors may also be common causes of both pain and higher symptom scores. The cross-sectional design cannot distinguish these possibilities.

This study also has some limitations. First, it relied on a self-reporting Internet-based survey conducted using a non-probability panel with quota sampling. Participation was limited to individuals registered in an online panel and accustomed to Internet surveys, which may have introduced selection bias. The research firm could not disclose the number of invitations and incomplete responses or the response rate, so selection bias due to non-participation cannot be evaluated; 753 responses that did not pass the research firm’s data quality checks (the detailed thresholds were not available to us) were also excluded. Second, as this was a questionnaire survey, data on climacteric symptoms and pain were based on subjective ratings, which may share method bias (common-method bias). Third, as this study only involved Japanese individuals, the generalizability of the results to other populations may be limited. Furthermore, cultural differences in social roles and stress must be considered when contemplating the relationship between psychological symptoms and pain in men. Fourth, important confounders were not measured: menopausal status, hormone therapy, medications (analgesics, antidepressants, and testosterone), comorbidities (e.g., osteoarthritis, depression, anxiety), sleep, occupation, income, education, exercise, and smoking; only age and BMI were adjusted for, so residual confounding is likely. Fifth, the SMI and AMS include items that overlap with pain, which may cause circularity; although we excluded these items in sensitivity analyses, items related to fatigue, sleep, and mood remained, and the shortened subscales (e.g., the SMI somatic subscale was reduced to a single item) may have lower reliability. The EQ-5D-5L also includes a pain/discomfort dimension, so its lower values at higher symptom scores partly reflect this overlap; it showed a ceiling effect (median 1.00 in the groups without pain and in Q1) and was analyzed only descriptively, using rank-based tests. Sixth, the SMI and AMS are not directly comparable (different items, score ranges, and subscale structure; vasomotor symptoms only in the SMI and sexual symptoms only in the AMS), so sex comparisons were made on within-sex z-scores and are only approximate. For the same reason, the symptom score quartiles were defined within each sex; they are relative groups and do not represent clinical severity. Seventh, pain was defined as an NRS score of 1 or higher over the previous 28 days, without information on duration, cause, or type (e.g., headache, dysmenorrhea, injury), so the findings cannot be applied to chronic pain. Eighth, pain intensity and pain locations were analyzed only in participants with pain (conditional analysis), which truncates the intensity distribution. Ninth, many statistical comparisons were performed, and the analyses are exploratory. With a large sample, small associations can be statistically significant; we therefore emphasized effect sizes and 95% CIs.

## 5. Conclusions

This study suggests that self-reported climacteric symptoms were associated with self-reported pain intensity in both middle-aged women and men. There was no clear evidence that the association differed by sex. Because of the cross-sectional design, overlapping items, unmeasured confounders, and the lack of hormone measurements, causal relationships and treatment implications cannot be inferred. Prospective studies that include hormone levels, menopausal status, and pain duration are warranted.

## Figures and Tables

**Table 1:** Characteristics of participants.

	Total	No Pain	Pain	*p*-Value No Pain vs. Pain
Women	*N* = 3000	*N* = 2567	*N* = 433	
Age (yr)	44.5 (37–52)	44 (37–52)	47 (39–54)	<0.001
BMI (kg/m^2^)	20.4 (18.8–22.7)	20.3 (18.8–22.6)	21.0 (19.1–23.3)	0.001
Pain intensity (NRS)			6 (5–7) *	
SMI total score	30 (12–49)	27 (9–46)	50 (33–66)	<0.001
SMI sub-scores				
vasomotor	11 (0–19)	9 (0–18)	18 (9–26)	<0.001
psychological	12 (3–21)	11 (0–19)	22 (12–29)	<0.001
somatic	6 (3–10)	6 (3–10)	10 (8–14)	<0.001
EQ-5D-5L	1.00 (0.82–1.00)	1.00 (0.87–1.00)	0.82 (0.74–0.89)	<0.001
**Men**	***N* = 3000**	***N* = 2512**	***N* = 488**	
Age (yr)	44.5 (37–52)	44 (37–52)	47 (40–54)	<0.001
BMI (kg/m^2^)	22.7 (20.7–25.1)	22.7 (20.7–25.1)	22.7 (20.7–25.5)	0.311
Pain intensity			5 (4–7)	
AMS total score	24 (18–36)	22 (17–33)	36 (28–49)	<0.001
AMS sub-scores				
psychological	6 (5–10)	5 (5–9)	10 (6.8–14.3)	<0.001
somatic	10 (7–15)	9 (7–14)	16 (12–21)	<0.001
sexual	6.5 (5–11)	6 (5–10)	10 (7–14)	<0.001
EQ-5D-5L	1.00 (0.82–1.00)	1.00 (0.87–1.00)	0.82 (0.68–0.89)	<0.001

Value: median (first and third quartiles). BMI: body mass index, SMI: the Simplified Menopausal Index, AMS: the Aging Males’ Symptoms, EQ-5D-5L: EuroQol-5 Dimensions-5 Levels, *: vs. Men: *p* < 0.01.

**Table 2:** Comparison of Pain Symptoms by Quartile Group of Climacteric Symptoms in Men and Women.

	Women	Men	*p*-Value
**Pain prevalence**			
Q1	2.8%	1.8%	0.249
Q2	10.1%	11.0%	0.597
Q3	15.3%	22.3%	0.001
Q4	30.0%	33.1%	0.195
**Pain intensity**			
Q1	4 (2–6)	4 (2–5)	0.823
Q2	5 (3–6)	4 (3–5.25)	0.234
Q3	5 (3–7)	5 (3–6)	0.020
Q4	6 (5–7)	6 (5–7)	0.063
**Number of pain locations**			
Q1	1 (1–2)	1 (1–1)	0.620
Q2	2 (1–2.25)	1 (1–2)	0.035
Q3	2 (1–3)	2 (1–3)	0.202
Q4	3 (2–4)	2 (1–3)	<0.001

Value: percentage or median (first and third quartiles).

**Table 3:** Correlation between pain intensity and climacteric symptoms (SMI or AMS) among participants with pain.

	Correlation Coefficient	*p*-Value
**Women (SMI)**		
Total score	0.352	<0.001
Sub-score		
vasomotor	0.299	<0.001
psychological	0.290	<0.001
somatic	0.331	<0.001
**Men (AMS)**		
Total score	0.387	<0.001
Sub-score		
psychological	0.376	<0.001
somatic	0.384	<0.001
sexual	0.265	<0.001

SMI: the Simplified Menopausal Index; AMS: the Aging Males’ Symptoms.

**Table 4:** Relationship between climacteric symptoms sub-score and pain intensity among participants with pain: multiple regression analysis.

	R^2^ /adj. R^2^	F	*p*	B	SE	β	*p*-Value	95%CI (B)		VIF
								Low	High	
**Women (SMI)** *N* = 433	0.145 /0.135	F (5,427) = 14.47	<0.001							
vasomotor				0.028	0.010	0.160	0.004	0.009	0.047	1.55
psychological				0.013	0.011	0.074	0.238	−0.009	0.035	1.97
somatic				0.112	0.031	0.208	<0.001	0.051	0.172	1.64
age				−0.004	0.010	−0.016	0.726	−0.023	0.016	1.04
BMI				0.035	0.022	0.072	0.109	−0.008	0.078	1.00
**Men (AMS)** *N* = 488	0.171 /0.162	F (5,482) = 19.88	<0.001							
psychological				0.074	0.027	0.203	0.005	0.022	0.127	3.07
somatic				0.071	0.023	0.224	0.002	0.025	0.117	3.15
sexual				0.005	0.024	0.012	0.836	−0.043	0.053	2.03
age				0.001	0.010	0.003	0.939	−0.019	0.021	1.07
BMI				0.002	0.021	0.004	0.916	−0.040	0.044	1.04

SMI: the Simplified Menopausal Index, AMS: the Aging Males’ Symptoms, B: non-standardized coefficient, β: Standardized coefficient, VIF: variance inflation factor.

**Table 5:** Sex differences in the associations between psychological and somatic subscores and pain intensity: pooled model with sex × subscore interaction terms.

Subscore	Women B (95% CI)	Men B (95% CI)	Interaction B (95% CI)	*p*-Value
Psychological	0.15 (−0.10 to 0.40)	0.35 (0.11 to 0.58)	0.20 (−0.14 to 0.54)	0.246
Somatic	0.52 (0.24 to 0.79)	0.40 (0.14 to 0.66)	−0.12 (−0.50 to 0.27)	0.546

B is the difference in pain intensity (numerical rating scale, 0–10) per 1-SD higher subscore, from a single multiple regression model in which the psychological and somatic subscores were entered simultaneously. Subscores of the SMI (women) and AMS (men) were standardized (z-scores) within each sex using all 3000 participants of that sex. The model also included sex, the sex-specific vasomotor (women) and sexual (men) scores, age, and body mass index. Interaction = difference in slope (men − women), tested with a sex × subscore term; sex-specific slopes were obtained by changing the reference category of sex. Vasomotor and sexual scores have no counterpart in the other sex and were not compared between sexes. CI: confidence interval; SMI: Simplified Menopausal Index; AMS: Aging Males’ Symptoms scale.

## Data Availability

The original data presented in the study, together with a data dictionary and the survey item wording, are openly available in Zenodo at https://doi.org/10.5281/zenodo.22931232 (all versions; the latest version resolves automatically).

## Supplementary Materials

The following supporting information can be downloaded at https://www.mdpi.com/article/10.3390/healthcare14213595/s1. Table S1. Characteristics of participants, Table S2. Prevalence of pain by symptom quartile group, age group (Panel A), and pain site (Panel B), Table S3. Correlation between pain intensity and climacteric symptoms (SMI or AMS) among participants with pain, Table S4. Association between total symptom score and pain intensity: pooled model with sex interaction (original and excluding pain-related items). Table S5. Relationship between climacteric symptoms sub-score and pain intensity among participants with pain: multiple regression analysis.

## Abbreviations

The following abbreviations are used in this manuscript:

SMI	Simplified Menopausal Index
AMS	Aging Males’ Symptoms
EQ-5D-5L	EuroQol-5 Dimensions-5 Levels
HRQOL	Health-related quality of life
NRS	Numerical Rating Scale
