# Supplementary material for: Association Between Self-Reported Climacteric Symptoms and Pain Intensity: A Cross-Sectional Study of 6000 Japanese Men and Women
Source: Healthcare (Basel). 2026 Oct 23;14(21):3595. doi: 10.3390/healthcare14213595 (PMC13763245; doi:10.3390/healthcare14213595)
Supplement: Supplementary file 1 [file healthcare-14-03595-s001.zip › healthcare-4614617-supplementary.pdf]

Supplementary Table S1. Characteristics of participants

	total	no pain	pain	P-value no pain vs pain
Women	N = 3,000	N = 2,567	N =433	
SMI total score	25 (9-41)	22 (7-38)	41 (26-55)	<0.001
SMI sub-scores				
vasomotor	-	-	-	-
psychological	10 (0-18)	9 (0-17)	18 (9-25)	<0.001
somatic (fatigue)	3 (0-5)	3 (0-5)	5 (3-7)	<0.001
Men	N = 3,000	N = 2,512	N = 488	
AMS total score	22 (16-34)	20 (16-31)	33 (25.6-46)	<0.001
AMS sub-scores				
psychological	-	-	-	-
somatic	9 (6-13)	8 (6-12)	14 (10-18)	<0.001
sexual	-	-	-	-

Value: median (first and third quartiles)

SMI: the Simplified Menopausal Index, AMS: the aging Males' Symptoms, -: Since it does not include the excluded items, it is the same as the original value.

In women, the somatic items include only fatigue, and the psychological items exclude headaches

Supplementary Table S2. Prevalence of pain by symptom quartile group, age group (Panel A), and pain site (Panel B)

Panel A. Age Group

Women (SMI)	Q1	Q2	Q3	Q4
30–34 yr.	7/145, 4.8 (2.4–9.6)	4/99, 4.0 (1.6–9.9)	18/120, 15.0 (9.7–22.5)	35/136, 25.7 (19.1–33.7)
35–39 yr.	0/106, 0.0 (0.0–3.5)	9/140, 6.4 (3.4–11.8)	10/123, 8.1 (4.5–14.3)	29/131, 22.1 (15.9–30.0)
40–44 yr.	3/127, 2.4 (0.8–6.7)	10/121, 8.3 (4.6–14.5)	11/119, 9.2 (5.2–15.8)	39/133, 29.3 (22.3–37.6)
45–49 yr.	5/125, 4.0 (1.7–9.0)	19/140, 13.6 (8.9–20.2)	17/125, 13.6 (8.7–20.7)	37/110, 33.6 (25.5–42.9)
50–54 yr.	1/128, 0.8 (0.1–4.3)	13/109, 11.9 (7.1–19.3)	29/136, 21.3 (15.3–28.9)	44/127, 34.6 (26.9–43.3)
55–59 yr.	5/131, 3.8 (1.6–8.6)	21/142, 14.8 (9.9–21.6)	29/123, 23.6 (16.9–31.8)	38/104, 36.5 (27.9–46.1)
Men (AMS)	Q1	Q2	Q3	Q4
30–34 yr.	2/193, 1.0 (0.3–3.7)	5/112, 4.5 (1.9–10.0)	19/88, 21.6 (14.3–31.3)	22/107, 20.6 (14.0–29.2)
35–39 yr.	5/162, 3.1 (1.3–7.0)	12/92, 13.0 (7.6–21.4)	17/115, 14.8 (9.4–22.4)	33/131, 25.2 (18.5–33.3)
40–44 yr.	1/148, 0.7 (0.1–3.7)	8/103, 7.8 (4.0–14.6)	31/133, 23.3 (16.9–31.2)	41/116, 35.3 (27.2–44.4)
45–49 yr.	1/148, 0.7 (0.1–3.7)	8/104, 7.7 (3.9–14.4)	23/115, 20.0 (13.7–28.2)	48/133, 36.1 (28.4–44.5)
50–54 yr.	3/139, 2.2 (0.7–6.2)	14/104, 13.5 (8.2–21.3)	35/140, 25.0 (18.6–32.8)	49/117, 41.9 (33.3–50.9)
55–59 yr.	5/133, 3.8 (1.6–8.5)	21/103, 20.4 (13.7–29.2)	41/153, 26.8 (20.4–34.3)	44/111, 39.6 (31.0–48.9)

Values are n with pain/N (%) with 95% confidence intervals (Wilson score method).

SMI: the Simplified Menopausal Index, AMS: the aging Males' Symptoms

Panel B. Pain Site

Women (SMI)	Q1 (N = 762)	Q2 (N = 751)	Q3 (N = 746)	Q4 (N = 741)
Head	3, 0.4 (0.1–1.2)	18, 2.4 (1.5–3.8)	28, 3.8 (2.6–5.4)	105, 14.2 (11.8–16.9)
Neck/shoulder	8, 1.0 (0.5–2.1)	36, 4.8 (3.5–6.6)	66, 8.8 (7.0–11.1)	145, 19.6 (16.9–22.6)
Back/low back	2, 0.3 (0.1–1.0)	37, 4.9 (3.6–6.7)	59, 7.9 (6.2–10.1)	135, 18.2 (15.6–21.2)
Abdomen	0, 0.0 (0.0–0.5)	11, 1.5 (0.8–2.6)	15, 2.0 (1.2–3.3)	55, 7.4 (5.7–9.5)
Upper extremity	5, 0.7 (0.3–1.5)	16, 2.1 (1.3–3.4)	37, 5.0 (3.6–6.8)	90, 12.1 (10.0–14.7)
Lower extremity	1, 0.1 (0.0–0.7)	30, 4.0 (2.8–5.6)	44, 5.9 (4.4–7.8)	121, 16.3 (13.8–19.2)
Others	3, 0.4 (0.1–1.2)	4, 0.5 (0.2–1.4)	9, 1.2 (0.6–2.3)	36, 4.9 (3.5–6.7)
**Men (AMS)**	**Q1 (N = 923)**	**Q2 (N = 618)**	**Q3 (N = 744)**	**Q4 (N = 715)**
Head	1, 0.1 (0.0–0.6)	2, 0.3 (0.1–1.2)	28, 3.8 (2.6–5.4)	55, 7.7 (6.0–9.9)
Neck/shoulder	4, 0.4 (0.2–1.1)	25, 4.0 (2.8–5.9)	86, 11.6 (9.5–14.1)	117, 16.4 (13.8–19.3)
Back/low back	5, 0.5 (0.2–1.3)	37, 6.0 (4.4–8.1)	83, 11.2 (9.1–13.6)	110, 15.4 (12.9–18.2)
Abdomen	0, 0.0 (0.0–0.4)	5, 0.8 (0.3–1.9)	18, 2.4 (1.5–3.8)	29, 4.1 (2.8–5.8)
Upper extremity	1, 0.1 (0.0–0.6)	11, 1.8 (1.0–3.2)	47, 6.3 (4.8–8.3)	68, 9.5 (7.6–11.9)
Lower extremity	7, 0.8 (0.4–1.6)	25, 4.0 (2.8–5.9)	67, 9.0 (7.2–11.3)	119, 16.6 (14.1–19.6)
Others	3, 0.3 (0.1–1.0)	6, 1.0 (0.4–2.1)	17, 2.3 (1.4–3.6)	45, 6.3 (4.7–8.3)

Values are n, % (95% CI, Wilson score method). Denominators are all participants in each quartile group. Participants could report pain at more than one site.

Supplementary Table S3. Correlation between pain intensity and climacteric symptoms (SMI or AMS) among participants with pain

	Pain intensity	
	Correlation Coefficient	p-value
Women (SMI)		
Total score	0.333	<0.001
Sub-score		
vasomotor	-	-
psychological	0.270	<0.001
somatic (fatigue)	0.262	<0.001
Men (AMS)		
Total score	0.377	<0.001
Sub-score		
psychological	-	-
somatic	0.367	<0.001
sexual	-	-

SMI: the Simplified Menopausal Index, AMS: the aging Males' Symptoms, -: Since it does not include the excluded items, it is the same as the original value.

In women, the somatic items include only fatigue, and the psychological items exclude headaches

Supplementary Table S4. Association between total symptom score and pain intensity: pooled model with sex interaction (original and excluding pain-related items)

Total score	Women B (95% CI)	Men B (95% CI)	Interaction B (95% CI)	p
Original (all items)	0.73 (0.54 to 0.91)	0.73 (0.58 to 0.88)	0.00 (−0.23 to 0.24)	0.991
Excluding pain-related items	0.66 (0.48 to 0.84)	0.71 (0.56 to 0.85)	0.04 (−0.19 to 0.27)	0.718

n = 921 participants with pain (433 women, 488 men). B is the difference in pain intensity (NRS) per 1-SD higher total score (z-score standardized within each sex), adjusted for age and BMI. Interaction = difference in slope (men − women) from the pooled model including a sex × z-score term. Sex-specific slopes were obtained by changing the reference category of sex. Excluded items: SMI, "shoulder stiffness, lumbago, or joint pain" and "headache, vertigo, or nausea"; AMS, "joint pain and muscular ache".

Supplementary Table S5. Relationship between climacteric symptoms sub-score and pain intensity among participants with pain: multiple regression analysis

	R^2 /adj. R^2	F	p	B	SE	β	p	95%CI（B）		VIF
								low	high	
Women (SMI) N=433	0.122 /0.111	F (5,427) =11.82	<0.001							
vasomotor				0.034	0.010	0.193	<0.001	0.015	0.053	1.49
psychological				0.019	0.012	0.093	0.130	-0.006	0.043	1.83
somatic (fatigue)				0.104	0.049	0.121	0.035	0.008	0.201	1.58
age				0.001	0.010	0.004	0.933	-0.019	0.021	1.02
BMI				0.037	0.022	0.076	0.096	-0.007	0.080	1.00
Men (AMS) N=488	0.163 /0.154	F (5,482) =18.80	<0.001							
psychological				0.087	0.027	0.238	0.002	0.033	0.141	3.21
somatic				0.059	0.027	0.166	0.031	0.005	0.112	3.40
sexual				0.011	0.024	0.027	0.654	-0.037	0.059	2.06
age				0.001	0.010	0.005	0.906	-0.019	0.021	1.07
BMI				0.005	0.022	0.009	0.826	-0.038	0.047	1.04

SMI: the Simplified Menopausal Index, AMS: the aging Males' Symptoms, B: non-standardized coefficient, β: Standardized coefficient, VIF: variance inflation factors
